# High expression of NQO1 is associated with poor prognosis in serous ovarian carcinoma

**DOI:** 10.1186/s12885-015-1271-4

**Published:** 2015-04-09

**Authors:** Xuelian Cui, Lianhua Li, Guanghai Yan, Kai Meng, Zhenhua Lin, Yunze Nan, Guang Jin, Chunyu Li

**Affiliations:** 1 Department of Pathology, Yanbian University Medical College, Yanji, 133002 China; 2 Cancer Research Center, Yanbian University, Yanji, 133002 China; 3 Department of Gynecology & Obstetrics, Yanbian University Hospital, Yanji, 133000 China

**Keywords:** Ovarian carcinoma, NQO1, Immunohistochemistry, Survival analysis

## Abstract

**Background:**

NAD(P)H:quinone oxidoreductase (NQO1) is a flavoprotein that catalyzes two-electron reduction and detoxification of quinones and its derivatives. NQO1 catalyzes reactions that have a protective effect against redox cycling, oxidative stress and neoplasia. High expression of NQO1 is associated with many solid tumors including those affecting the colon, breast and pancreas; however, its role in the progression of ovarian carcinoma is largely undefined. This study aimed to investigate the clinicopathological significance of high NQO1 expression in serous ovarian carcinoma.

**Methods:**

NQO1 protein expression was assessed using immunohistochemical (IHC) staining in 160 patients with serous ovarian carcinoma, 62 patients with ovarian borderline tumors and 53 patients with benign ovarian tumors. Quantitative real-time polymerase chain reaction (qRT-PCR) was performed to detect NQO1 mRNA expression levels. The correlation between high NQO1 expression and clinicopathological features of ovarian carcinoma was evaluated by Chi-square and Fisher’s exact test. Overall survival (OS) rates of all of ovarian carcinoma patients were calculated using the Kaplan-Meier method, and univariate and multivariate analyses were performed using the Cox proportional hazards regression model.

**Results:**

NQO1 protein expression in ovarian carcinoma cells was predominantly cytoplasmic. Strong, positive expression of NQO1 protein was observed in 63.8% (102/160) of ovarian carcinomas, which was significantly higher than in borderline serous tumors (32.3%, 20/62) or benign serous tumors (11.3%, 6/53). Importantly, the rate of strong, positive NQO1 expression in borderline serous tumors was also higher than in benign serous tumors. High expression of NQO1 protein was closely associated with higher histological grade, advanced clinical stage and lower OS rates in ovarian carcinomas. Moreover, multivariate analysis indicated that NQO1 was a significant independent prognostic factor, in addition to clinical stage, in patients with ovarian carcinoma.

**Conclusions:**

NQO1 is frequently upregulated in ovarian carcinoma. High expressin of NQO1 protein may be an effective biomarker for poor prognostic evaluation of patients with serous ovarian carcinomas.

## Background

Ovarian carcinoma is one of the most lethal gynecological carcinomas, and the second leading cause of carcinoma-related deaths among women [1]. The majority of ovarian carcinoma cases are of epithelial origin (> 90%), and of these, serous ovarian carcinoma is the most common subtype [2]. As ovarian carcinoma is often asymptomatic in the early stages, or presents with vague symptoms mimicking extra-ovarian disease, the majority of patients (70–75%) present with widespread disease at diagnosis, and as such the mortality rate is high [3]. Therefore, understanding the molecular pathogenesis and mechanisms underlying ovarian cancer initiation and progression is critical for the prevention and treatment of this disease.

The NAD(P)H:quinone oxidoreductase 1 (NQO1) is a predominantly cytosolic enzyme, which uses NADH or NADPH as substrates to directly reduce quinones to hydroquinones [4]. The direct two-electron reduction of quinones to hydroquinone by NQO1 is historically considered a detoxification mechanism, since this reaction bypasses the formation of the highly reactive semiquinone [5]. NQO1 provides cells with multiple layers of protection against oxidative stress, including the direct detoxification of highly reactive quinones, the maintenance of lipid-soluble antioxidants in reduced forms, and stabilization of the tumor suppressor p53 [6]. Chronic inflammation suppresses NQO1 expression and may increase susceptibility to cell injury. Paradoxically, increasing evidence suggests that high expression of NQO1 at the early stages of carcinogenesis may provide cancer cells with a growth advantage [7-9]. Moreover, certain quinones, such as mitomycin C, streptonigrin, E09 and RH1, are bioactivated by NQO1 [10-14]. The bioactivation property of NQO1 renders it an ideal target for developing anti-tumor drugs, since NQO1 activities are elevated in various human tumors [15]. NQO1 was shown to be expressed at high levels in many solid tumors, including uterine cervix [16], lung [17] and pancreas [18], and was also detected following the induction of cell cycle progression and proliferation of melanoma cells [19]. However, to date, the role of NQO1 in ovarian carcinoma progression remains unclear.

In the present study, we investigated the correlation between high expression of NQO1 and clinicopathological features of serous ovarian carcinomas. We also assessed the prognostic value of high NQO1 expression in patients with serous ovarian carcinoma.

## Methods

### Ethics statement

This study complied with the Helsinki Declaration and was approved by the Human Ethics and Research Ethics committees of Yanbian University Medical College of China. Through the surgery consent form, patients were informed that the resected specimens were stored by our hospital and potentially used for scientific research, and that their privacy would be maintained. Follow-up survival data were collected retrospectively through medical-record analyses.

### Patients and tissue specimens

A total of 275 human ovarian tumor specimens, including 160 serous carcinomas, 62 borderline serous tumors, 53 benign serous tumors were used for this study. These tumors were selected randomly from patients undergoing surgery between 2005 and 2010 and stored in the Tumor Tissue Bank of Yanbian University Medical College. Pathological parameters, including age, menopausal status, grade and survival data, were carefully reviewed in all 160 serous ovarian carcinomas.

In 160 cases, patients’ ages ≥ 48 years to <48 years was 97:63. The hematoxylin and eosin-stained slides of the different biopsies were reviewed by two experienced pathologists and one appropriate paraffin block was selected for this study. Histopathological grades were made using the World Health Organization (Pathology & Genetics Tumors of gynecological system) criteria. All of the ovarian carcinoma patients were clinically staged according to the FIGO staging system [with 92 early- stage tumors (FIGO stages I and II) and 68 late-stage tumors (FIGO stages III and IV)]. None of the ovarian carcinoma patients received preoperative radiation or chemotherapy before surgery. All ovarian carcinoma patients had follow-up records for more than 5 years. By February 2014, 111 patients had died while 49 patients remained alive. The median survival time was 44.5 months.

### Immunohistochemical (IHC) analysis

IHC analysis was performed using the DAKO LSAB kit (DAKO A/S, Glostrup, Denmark). Briefly, to eliminate endogenous peroxidase activity, 4 μm thick tissue sections were deparaffinized, rehydrated and incubated with 3% H_2_O_2_ in methanol for 15 min at room temperature (RT). The antigen was retrieved at 95°C for 20 min by placing the slides in 0.01 M sodium citrate buffer (pH 6.0). The slides were then incubated with the NQO1 antibody (1:200, Santa Cruz Biotechnology, Dallas, TX, USA) at 4°C overnight. After incubation with the biotinylated secondary antibody at RT for 30 min, the slides were incubated with streptavidin-peroxidase complex at RT for 30 min. Immunostaining was developed by using 3,3′-diaminobenzidine and Mayer’s hematoxylin was used for counterstaining [16]. Mouse IgG was used as an isotype control. In addition, positive tissue sections were processed while omitting the primary antibody (mouse anti-NQO1) as negative controls.

### Evaluation of the IHC staining

As described previously [20], the expressions were scored by two pathologists (Lin Z & Liu S) who did not possess knowledge of the clinical data. In case of discrepancies, a final score was established by reassessment on a double-headed microscope. Briefly, the immunostaining for NQO1 was semi-quantitatively scored as ‘-’ (negative, no or less than 5% positive cells), ‘+’ (5–25% positive cells), ‘++’ (26–50% positive cells) and ‘+++’ (more than 50% positive cells). Only the cytoplasmic expression pattern was considered as positive staining. Tissue sections scored as ‘++’ and ‘+++’ were considered as strong positives (high expression) of NQO1. For survival data analysis, ‘++’ or ‘+++’ scored samples were considered as high NQO1 expression and ‘-’or ‘+’ scored samples were considered as low NQO1 expression.

### qRT-PCR

Total RNA was extracted using TRIzol Reagent (Invitrogen, Carlsbad, CA, USA) from fresh tissue samples of 19 serous ovarian carcinomas and 15 benign serous ovarian tumors. First-strand cDNA was synthesized by PrimeScript reverse transcriptase (TaKaRa Biotechnology, Dalian, China) and oligo (dT) following the manufacturer’s instructions. Real-time PCR was performed using double-stranded DNA-specific SYBR Premix Ex Taq™ II Kit (TaKaRa Biotechnology) on a Bio-Rad sequence detection system according to the manufacturer’s instructions. Double-stranded DNA specific expression was tested by the comparative Ct method using 2^-ΔΔCt^. NQO1 primers were as follows: 5′-GGCAGAAGAGCACTGATCGTA-3′, and 5′-TGATGGGATTGAAGTTCATGGC-3′. Primers for GAPDH, which was used as an internal control, were: 5′-GGTCTCCTCTGACTTCAACA-3′ and 5′-ATACCAGGAAATGAGCTTGA-3′. All assays were performed at least three times.

### Statistical analysis

Statistical analyses included descriptive statistics with determination of minimal and maximal values, means and medians, with 95% confidence interval (CI) for particular variables. Chi-square test and Fisher’s exact test were used to assess correlation between clinicopathological characteristics and the expression of studied protein. Kaplan-Meier method was used to calculate the survival rates after tumor removal and Log-rank was used to analyze the differences in survival curves. Multivariate survival analysis was performed on all the significant characteristics measured by univariate survival analysis (age, menopausal status, histological grade, FIGO stage and NQO1 expression) through the Cox proportional hazard regression model. Statistical calculation was performed using the SPSS 17.0. *P* < 0.05 was considered statistically significant.

## Results

### High expression of NQO1 protein in serous ovarian carcinoma

**Figure 1:**
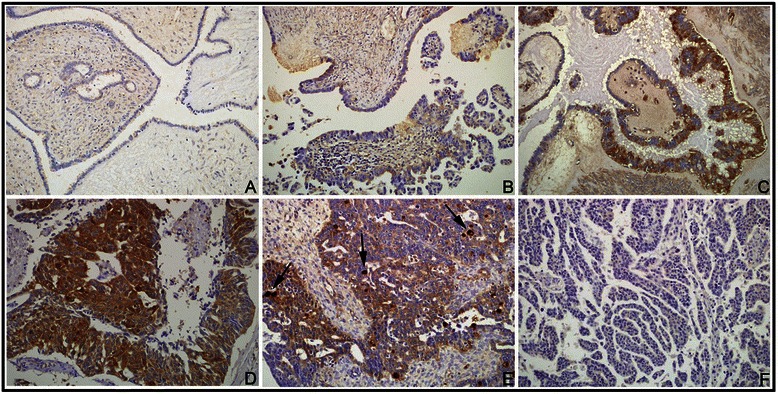
**IHC staining of NQO1 protein in ovarian tumor samples. (A)** Negative expression of NQO1 protein in a benign serous tumor. **(B–C)** Weak positive expression of NQO1 protein (B) and positive expression (C) in atypical cells of borderline serous tumors. **(D)** Strong positive expression of NQO1 protein in serous carcinoma cells, in a patient with metastasis. **(E)** Positive expression of NQO1 protein in a serous carcinoma patient without metastasis. Scattered, strongly positive-staining cancer cells are seen (*arrows*). **(F)** Negative expression of NQO1 protein in a serous carcinoma patient without metastasis. Original magnification, A–F: ×200.

IHC analysis of NQO1 in ovarian carcinoma cells from 160 patients revealed predominantly cytoplasmic expression (Figure 1). The rate of positive NQO1 protein expression was significantly higher in serous carcinomas (85.6%, 137/160) than in borderline serous tumors (56.5%, 35/62) or benign serous tumors (34.0%, 18/53) (*P* < 0.01, respectively). Similarly, the rate of strong, positive NQO1 protein expression was significantly higher in serous carcinomas (63.8%, 102/160) than in either borderline serous tumors (32.3%, 20/62) or benign serous tumors (11.3%, 6/53) (*P* < 0.01, respectively). More importantly, the rates of positive and strongly positive NQO1 protein expression in borderline serous tumors were significantly higher than in benign serous tumors (*P* < 0.05) (Table 1).

**Table 1:** **NQO1 expression in ovarian carcinomas**

Diagnosis	No. of cases	Positive cases				Positive cases rates	Strongly positive rates
		-	+	++	+++		
Serous carcinoma	160	23	35	67	35	85.6%**^#^	63.8%**^#^
Borderline serous tumor	62	27	15	12	8	56.5%*	32.3%*
Benign serous tumor	53	35	12	4	2	34.0%	11.3%

*P < 0.05, **P < 0.01, compared with benign serous tumor.

#P < 0.01, compared with borderline serous tumor.

**Figure 2:**
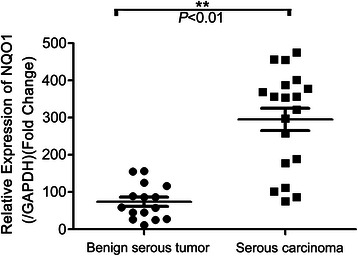
**qRT-PCR analysis of NQO1 mRNA.** Serous carcinoma specimens (n = 19) and benign serous tumors (n = 15) were collected, and NQO1 mRNA levels were assessed by qRT-PCR. Error bars represent the standard deviation of the mean (SD) calculated from three parallel experiments. **P < 0.01.

In keeping with these results, analysis of NQO1 mRNA levels by qRT-PCR confirmed elevated levels of NQO1 transcript in serous ovarian carcinoma samples compared with benign ovarian tumors in fresh tissues (Figure 2).

### Correlation between NQO1 expression status and clinicopathological features of serous ovarian carcinoma

To evaluate the relationship between NQO1 protein and ovarian carcinoma progression, we analyzed the correlation between high NQO1 expression and clinicopathological features of ovarian carcinomas. The strongly positive rates of NQO1 protein were significantly higher in Grade 2 (G2) (61.7%, 29/47) and Grade 3 (G3) (69.7%, 53/76) ovarian carcinomas than those in Grade 1 (G1) (27.0%, 10/37) cases (*P* = 0.000). For the FIGO clinical stages, the strongly positive rate of NQO1 protein was 80.9% (55/68) in the late-stage (IIB–IIIC) ovarian carcinomas, but only 40.2% (37/92) in early-stage (I–IIA) cases (*P* = 0.000). However, high expression of NQO1 protein was not related with age, menopausal status of patients with ovarian carcinoma (Table 2).

**Table 2:** **Correlation between NQO1 protein expression and the clinicopathological parameters of ovarian carcinoma**

Variables	No. of cases	NQO1 strongly positive cases (%)	*χ* ^*2*^	*P*value
**Age**			0.161	0.689
≥48	97	57 (58.8%)		
<48	63	35 (55.6%)		
**Menopausal status**			0.050	0.823
Premenopausal	76	43 (56.6%)		
Postmenopausal	84	49 (58.3%)		
**Histological grade**			19.056	0.000**
Grade-1	37	10 (27.0%)		
Grade-2	47	29 (61.7%)		
Grade-3	76	53 (69.7%)		
**FIGO stage**			26.458	0.000**
I-II	92	37 (40.2%)		
III-IV	68	55 (80.9%)		

**P < 0.01.

### High NQO1 expression is an independent biomarker of poor prognosis in patients with serous ovarian carcinoma

**Figure 3:**
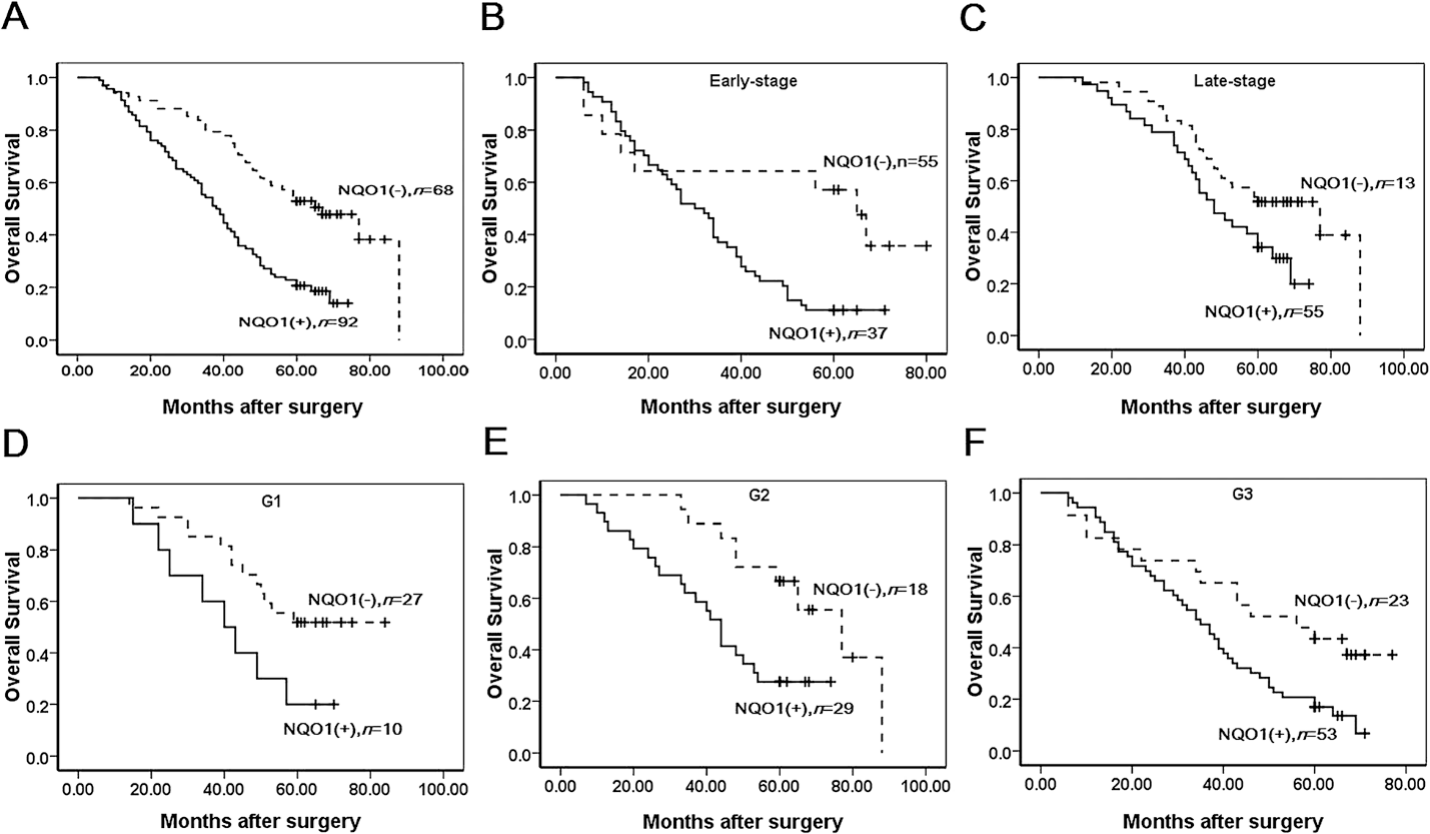
**Kaplan-Meier survival curves illustrating the significance of NQO1 expression in ovarian carcinomas. (A)** OS rates of patients with high (solid, n = 92) and low (dashed, n = 68) NQO1 expression. A: Log-rank = 21.699, *P* = 0.000. **(B**-**C)** High NQO1 expression was strongly associated with poor OS in early-stage (solid, n = 37) and late-stage (solid, n = 55). B: Log-rank = 6.527, P = 0.011; C: Log-rank = 4.806, *P* = 0.028. **(D-F)** High NQO1 expression was strongly associated with poor OS in G1 (solid, n = 10), G2 (solid, n = 29) and G3 (solid, n = 53). D: Log-rank = 4.359, *P* = 0.037; E: Log-rank = 7.020, *P* = 0.008; F: Log-rank = 5.978, *P* = 0.015).

To further substantiate the importance of high NQO1 expression in ovarian carcinoma progression, we analyzed the OS of 160 ovarian carcinoma patients using the Kaplan–Meier method. Patients with high NQO1 expression exhibited a lower rate of OS than those with low NQO1 expression (Log-rank = 21.699, *P* = 0.000) (Figure 3A). Similarly, ovarian carcinoma patients with high NQO1 expression had decreased OS compared with those with low NQO1 expression in either early-stage cases (Log-rank = 6.527, *P* = 0.011) or late-stage cases (Log-rank = 4.806, *P* = 0.028) (Figure 3B–C). Moreover, survival of patients with G1 (Log-rank = 4.359, *P* = 0.037), G2 (Log-rank = 7.020, *P* = 0.008) and G3 (Log-rank = 5.978, *P* = 0.015) ovarian carcinoma was significantly lower in patients with tumors exhibiting high versus low NQO1 expression (Figure 3D–F).

Univariate analysis demonstrated that histological grade (*P* = 0.020), FIGO stage (*P* = 0.000) and NQO1 expression status (*P* = 0.000) were all significantly associated with OS in patients with ovarian carcinoma. These data suggest that NQO1 may be a valuable prognostic factor in ovarian carcinoma. Multivariate analysis was subsequently performed using the Cox proportional hazards model for all significant variables examined in the univariate analysis. We found that high expression of NQO1 (HR: 1.796, 95% CI: 1.250–2.580, *P* = 0.002) and FIGO stage (HR: 1.736, 95% CI: 1.228–2.453, *P* = 0.002) were significant independent prognostic factors for survival in ovarian carcinoma (Table 3).

**Table 3:** **Univariate and multivariate analysis of clinicopathological factors for the overall survival rate of 160 patients with ovarian carcinoma**

Characteristics	Univariate analysis HR (95%CI)	*P*value	Multivariate analysis HR (95%CI)	*P*value
**Age**	1.339(0.964-1.860)	0.082	1.391(0.992-1.951)	0.056
**Menopausal status**	1.115(0.815-1.525)	0.496	1.177(0.842-1.646)	0.340
**Histological grade**	1.268(1.038-1.548)	0.020*	1.099(0.881-1.371)	0.404
**FIGO stage**	1.944(1.414-2.672)	0.000**	1.736(1.228-2.453)	0.002**
**NQO1**	2.167(1.555-3.021)	0.000**	1.796(1.250-2.580)	0.002**

HR: hazard ratio; CI: confidence interval.

*P < 0.05, **P < 0.01.

## Discussion

The catalytic properties of NQO1 were first reported by Ernster and Navazio in 1958 [21]. NQO1 is predominantly located in the cytoplasm, but low levels of NQO1 have also been identified in the nucleus under normal conditions [22]. Several studies have indicated that the phase II enzyme, NQO1, catalyzes the metabolic detoxification of quinones and protects cells against chemical-induced oxidative stress and cancer [23,24]. The importance of NQO1 in cancer prevention was supported by the finding that NQO1-null mice are more susceptible to 7,12-dimethylbenz(*a*)anthracene- and benzo(*a*)pyrene-induced skin cancer [25,26]. In contrast, Choi *et al.* demonstrated that inhibition of NQO1 activity decreases melanogenesis, whereas overexpression of NQO1 enhances pigmentation [27]. Studies using an NQO1 inhibitor suggest that this oxidoreductase plays a role in inducing the growth of pancreatic cells [28]. Beyond these reports, however, the function of NQO1 in tumor progression remains controversial.

To date, many studies have shown that polymorphisms in the *NQO1* gene affect the translation of the NQO1 protein. The NQO1 C609T polymorphism has been associated with an increased risk of various malignancies, including lung [29], esophageal [30], gastric [31] and uterine cervix [16] cancers. Goode *et al.* reported that an NQO1 single-nucleotide polymorphism (SNP) is associated with an increased risk of ovarian cancer [32]. Moreover, high NQO1 expression has been observed in many cancers of the liver, thyroid, breast, colon and pancreas. Siegel *et al.* also found that NQO1 was over-expressed in ovarian carcinoma compared with normal tissue [33]. However, to date, the role of NQO1 as a biomarker in ovarian carcinoma progression has not been elucidated.

Here, we performed IHC staining of NQO1 protein using 160 serous ovarian carcinomas, 62 borderline serous tumors and 53 benign serous tumors. We observed that expression of NQO1 protein (positive and strongly positive) was significantly higher in serous carcinomas compared with either borderline or benign serous tumors (*P* < 0.01). More importantly, we observed a significant difference in the rates of positive and strongly positive NQO1 expression between borderline serous tumors and benign serous tumors (*P* < 0.05), indicating that NQO1 may play an important role in the progression of ovarian carcinoma. Compatible with these findings, we also observed that the rate of strongly positive NQO1 protein expression was significantly higher in patients with late-stage serous ovarian carcinoma, compared with early-stage cases. Similarly, the rate of strongly positive NQO1 protein expression was higher in patients with G2 or G3, compared with G1 ovarian carcinomas. High-grade serous ovarian carcinoma is the most lethal form of gynecological malignant carcinoma, and the majority of patients present with late clinical stages (FIGO stages III and IV) of disease at the time of diagnosis. Our data also demonstrate that positive NQO1 expression is significantly correlated with high-grade and late-stage ovarian carcinoma. qRT-PCR analysis also confirmed increased levels of NQO1 mRNA in serous ovarian carcinoma samples compared with benign ovarian tumors in fresh tissues. These results indicate that NQO1 may be a useful biomarker for poor prognosis in patients with ovarian carcinoma.

Previously, we have shown that high expression of NQO1 protein was strongly associated with advanced stage, lymph node metastasis, Her2 overexpression and shortened survival of patients with breast cancer [34]. Moreover, we demonstrated that high expression of NQO1 in cervical squamous cell carcinoma patients was associated with lower disease-free survival (DFS) and 5-year OS rates compared with patients with low-level NQO1 expression [16]. Buranrat *et al.* also reported a significant association between high NQO1 expression and short overall survival in cholangiocarcinoma patients, raising the exciting possibility of using NQO1 as a tumor marker [35]. With respect to survival, we found that ovarian carcinoma patients exhibiting high NQO1 expression had lower OS rates compared with patients with low NQO1 expression (*P* < 0.01). Univariate survival analysis revealed that tumor histological grade, FIGO stage and NQO1 expression status were all significantly related to OS of patients with serous ovarian carcinoma (*P* < 0.05). Further multivariate survival analysis revealed that NQO1 expression was an independent prognostic factor, as was FIGO stage. Our clinical and experimental data indicate that NQO1 is a prognostic factor and a potential therapeutic target in patients with serous ovarian carcinoma.

Recently, NQO1 has been targeted in tumor cells, exemplifying an ‘enzyme directed’ approach to anticancer drug development [36]. Kung *et al.* demonstrated that β-Lapachone-induced cytotoxicity of three different lung cancer cell lines was positively correlated with NQO1 expression and enzyme activity [37]. Hadley *et al.* suggested that stratification of patients on the basis of NQO1 protein levels could identify a subset of esophageal squamous cell carcinomas patients that may potentially benefit from administration of low doses of 17-AAG, possibly in combination with other chemotherapeutics [38]. Huang *et al.* reported that the potency and NQO1-dependent therapeutic window of deoxynyboquinone and its apparent reduced metabolism by one-electron oxidoreductases make this drug (or derivatives) very promising [39]. Further studies are therefore necessary to verify whether NQO1 inhibitors may be of clinical benefit to patients with ovarian carcinoma.

## Conclusions

NQO1 is frequently upregulated in ovarian carcinoma, and its high expression predicts poor prognosis of patients with ovarian carcinoma. NQO1 may serve as a new prognostic factor and potential therapeutic target for patients with serous ovarian carcinoma.
